# Technical Modifications for the Application of the Total Difference Method for Frontal Sinus Comparison

**DOI:** 10.3390/biology11071075

**Published:** 2022-07-19

**Authors:** Jessica L. Campbell, Lauren N. Butaric

**Affiliations:** Department of Anatomy, Des Moines University, 3200 Grand Avenue, Des Moines, IA 50312, USA; lauren.butaric@dmu.edu

**Keywords:** frontal sinus, personal identification, radiographic comparison, forensic anthropology, shape outline analysis, ImageJ

## Abstract

**Simple Summary:**

The individuality observed in the frontal sinus cavity (located in the skull) can be used to corroborate an identification of an unknown skeleton, with a success rate that is comparable to that obtained by using dentition or DNA. For admissibility in court, however, forensic methods should be continually tested to assess their repeatability and accuracy. This study tests one technical method for obtaining the frontal sinus shape: the Total Difference Method, which quantifies the sinus shape from 59 linear measurements. We develop a more streamlined application by using a measurement aid (a tracing overlay) and a semi-automated macro to collect the multiple required measurements. The radiographs of 10 adults were assessed by two observers. The data were collected following the original method in addition to the use of a measurement aid with and without the semi-automated macro. The results indicate that this technique for assessing the frontal sinus is reliable and repeatable between and within observers, both for the original method and when using the newly developed applications. Further, the semi-automated macro improved the accuracy and efficiency over the original method. Importantly, the measurement aid will allow researchers to conduct the Total Difference Method on a larger scale, creating comparative databases that can be utilized for future forensic research and practice.

**Abstract:**

Despite being used in personal identification since the 1920s, frontal sinus-based methods are rarely validated. This study is a validation test of the Total Difference Method (TDM). The posterior-anterior radiographs of 10 adults were assessed by two observers using three modes: the Freehand Mode largely followed the original protocols; the Overlay Mode utilized a tracing overlay; and the Semi-Auto Mode used the overlay and macro, walking the user through multiple steps. The modes were evaluated for the time taken to complete each image and the accuracy and repeatability of the line lengths, midline assessment, and angle placement. The repeated measures analysis of variance results for the intra-observer error revealed differences in bias in the angle placement and line length mean error between the rounds and modes. The differences between the rounds were approximately consistent for each mode, suggesting observer error. Significant differences in the inaccuracy of the angle placement and the line lengths between observers by mode were evident; in post hoc testing, the Freehand Mode and Overlay Mode had the greatest error in both variables (*p.adj* < 0.0001). The Semi-Auto Mode retained no significant error for angle inaccuracy and had the fewest errors for line length inaccuracy (*p.adj* < 0.01). When using the Semi-Auto Mode, the time was 46.1% faster than that of the Freehand Mode and 34% faster than that of the Overlay Mode (F_2,18_ = 52.71, *p <* 0.0001); time slightly improved with method familiarity. The results suggest that the technique required for the TDM can reliably be repeated, and the semi-automated macro improves accuracy and efficiency, but only after the users are familiar with the method and macro procedures. All resources needed to repeat this study are freely available on GitHub.

## 1. Introduction

When faced with an unknown decedent, medical examiners, investigators, and forensic personnel are tasked with obtaining a positive identification as quickly as possible. Identifications by facial recognition and fingerprints require soft tissue preservation. Dental comparisons can be performed on decomposed or skeletal remains, but teeth are not recovered in all cases, and recent dental records are not always available. Although it is possible to obtain DNA from most remains, certain taphonomic processes can degrade DNA. DNA analyses are also expensive and can have long turnaround rates (at times, >1 year). Radiographic frontal sinus comparison is another possible method of identification if antemortem records are available, as the size and shape of the frontal sinus have been documented to be unique per individual. Even monozygotic twins present unique frontal sinus patterns [1,2], leading to their referral as the “fingerprints” of the skull [1,3,4]. With this in mind, the use of radiographic identification based on frontal sinus comparisons has been recognized since the 1920s, e.g., [2,5,6].

Many of these early forensic studies establishing frontal sinus “uniqueness” are based on subjective, anecdotal evidence, with conclusions drawn from simple visual comparisons between antemortem and postmortem radiographic images, e.g., [1,2,5,6,7]. While this visual-based method has a reportedly high rate of success [8,9,10,11], others suggest it lacks the objectivity and statistical rigor now expected for admissibility in court, as detailed by the *Daubert* guidelines [12] and the 2009 National Academy of Sciences report [13]. The *Daubert* guidelines for helping to assess whether a scientific method is admissible in court include that the method has: (1) been tested using the scientific method; (2) undergone peer review and publication; (3) documented error rates; (4) incorporated professional standards, and (5) received general acceptance by the relevant scientific community [14,15].

Several methods attempt to objectively quantify frontal sinus variation using metrics, code-based scoring systems, and/or outline analyses [9,16,17,18,19,20,21,22]. These methods typically utilize the unique anatomical characteristics of the frontal sinus, as seen in frontal radiographic views: presence/absence of the sinus; bilateral versus unilateral presentation of the right and left lobes; arcade (or scallop) number and distribution; morphological presentation of the intra- and intersinus septa; overall height-breadth dynamics; overall traced outline; etc. After collecting these variables, identification methods use various statistical techniques to determine the probability of whether the sinus in the postmortem image is a match (or a non-match) to that provided in the antemortem image. While numerous quantitative methods of frontal sinus identification have been developed, few have been rigorously tested for validity and repeatability. This is of utmost importance, as questions regarding the reliability/validity of a method are one of the most frequent reasons forensic testimony is thrown out [23].

One example of the outline methods in the forensic literature lacking validation is the Total Difference Method (TDM) developed by Cox et al. [16]. In this method, the frontal sinus outline is traced, and then 59 linear measurements are collected from the origin (cranial midline) to the sinus outline at 3° intervals. For each individual, these 59 linear measurements are standardized by their sinus baseline length. The “total difference” refers to the sum of each line’s absolute difference between an antemortem and postmortem pair of images. In theory, in an array of possible matches, the matching frontal sinus should have the lowest Total Difference, with the value approaching zero. While the original publication reported high repeatability and identification rates (correctly discriminating 100% of matching pairs and 97% of non-matching pairs), the authors cautioned that additional error rate testing is needed. Despite the potential ability of the TDM [16] to quantitatively and objectively evaluate frontal sinus comparisons, the method has not been externally validated. Further, based on our experience with the method, the process of manually collecting measurements from 59 three-degree intervals is time-consuming, since it must be completed in one sitting, and errors in line placement can be difficult to find or correct without starting anew.

This study attempted to address these concerns with the Cox et al. TDM [16] by first directly testing the repeatability of this method. In addition, this study develops and tests a more streamlined procedure to collect the necessary measures at 3° intervals. Specifically, we have developed a measurement aid (an overlay with 3° intervals from a central origin) and a semi-automated macro, with the goal of increasing efficiency and repeatability when validating the TDM. The results of this study will provide necessary documentation of the repeatability and error associated with the TDM for frontal sinus identification, as well as the developed products that will ultimately allow for future testing of this method using more robust sample sizes.

## 2. Materials and Methods

### 2.1. Sample

The current study utilizes posterior-anterior radiographs originating from the American Association of Orthodontists Foundation (AAOF) Craniofacial Growth Legacy Collections Project [24]. This freely available, online resource houses longitudinal radiographs from several growth series. To obtain the largest sample possible, the current study utilized radiographs from several collections: the Case Western Bolton-Brush Growth Study, the University of Toronto Burlington Growth Study, and the Oregon Growth Study. Although ancestry is not listed, the samples are likely largely composed of European-American individuals. Radiographs and frontal sinus outlines (see details below) were previously collected for larger, ongoing studies of frontal sinus morphology and identification methods, e.g., [25,26]. The sample included ten outlines which were randomly selected in R from 244 possible sinus outlines on 128 adult individuals used in a previous study (note: some individuals had several radiographs at different age points, explaining the higher number of outlines versus individuals) [27]. Note that, although the sample of 10 individuals seems small, each outline contributes a total of 59 linear measurements for a final sample size of 590 data points collected per mode. This sample size estimates a very small effect size for f tests (<0.25) given an alpha of 0.05, a power of 0.95 (probability of a false negative, beta = 0.05), and an assumed high correlation among repeated measures [28].

As a recent study noted that the frontal sinus shape stabilizes at around 15 years of age in females and 18 years of age in males [26], the inclusion criteria included an age of 18 years or older (adult) and no obvious pathological conditions. Sex was not considered in obtaining the random sample, as the original study did not find sex-based significant differences [16]. Following Cox et al. [16], individuals also had to have a frontal sinus extending superiorly above the supraorbital borders, localized over the midline of the cranium (i.e., not unilaterally distributed on one side), and with the right and left lobes touching (i.e., they could not be discontinuous). In total, 18 out of the 128 individuals (14%) exhibited sinuses that were discontinuous or unilateral and thus not appropriate for the TDM method. During the random sampling process, two discontinuous outlines were removed and replaced with the next random sequential outline. The final sample of 10 outlines included 10 unique individuals (6 females, 4 males), with no duplication of individual outlines. The individuals ranged from 18 to 26 years of age, with an average age of 19.7 years.

### 2.2. Data Collection

Each radiograph was downloaded and imported into the freeware program ImageJ [29] and its expansion FIJI [30] (hereby referenced together as “ImageJ”). While the use of ImageJ is a deviation from the original method’s selection of Photoshop^®^ CS2 [31], the tools in ImageJ are similar to the measurement and selection tools described in the method’s original publication and should render the same results. Further, this program is freely available, allowing for wider-spread access for researchers wishing to use or test this technique. Additional advantages to using this software, specific to the data collection methods used here, are outlined below.

As noted in the introduction, a primary component of this study was to create and test the use of measurement aids for the more efficient collection of the data required for the Total Difference Method. To this end, we collected data using three variations: (1) in the Freehand Mode, data were collected consistently with the original method (the exception being that data were captured in ImageJ) with no measurement aid; (2) the Overlay Mode applied a measurement aid (referred to as the “overlay”) to visualize the 3° rays around the sinus; and (3) while also utilizing the overlay, the Semi-Auto Mode applied a semi-automated macro that directed users through the steps of the method, including automatically saving the data and cycling through subsequent images. The specific protocols for each mode, and the variables collected, are described further below. The data were collected by two observers with professional-level experience in sinus assessment (L.N.B.) and personal identification (J.L.C.).

**Figure 1:**
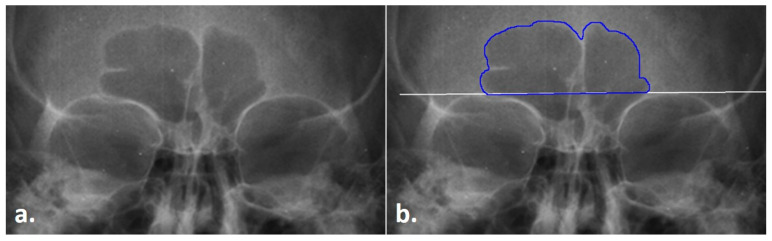
Radiograph of the frontal sinus; (**a**) untreated radiograph, (**b**) frontal sinus with the outline (blue) and supraorbital line (white).

Prior to collecting data in the three modes (see below), the first step of the TDM is to obtain the frontal sinus outline. In this step, .png radiographic images downloaded from the Legacy Collection were opened in ImageJ. The images were increased to 3000 pixels. Following Christensen [14,17] and Cox et al. [16], the inferior boundary of the frontal sinus was demarcated at the supraorbital line using the *line tool* in ImageJ. From this line, the lateral and superior edges of the sinus were traced from the left to the right side of the image (clockwise fashion) using the *Freehand tool* in ImageJ; note that only the outer contours were traced, and individual septa were not followed inferiorly into the sinus, following Christensen [14,17] (see Figure 1). To avoid potential inter-reliability errors in the sinus outlines not directly related to the TDM, the outlines were only traced by a single researcher. Each outlined image was saved as a .tiff file for future processing, as detailed below.

**Figure 2:**
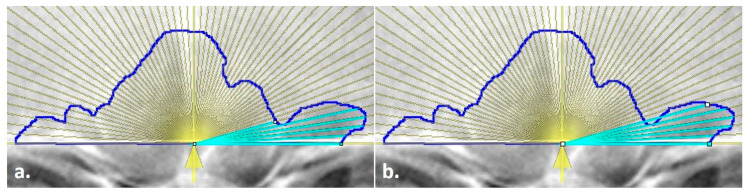
Line endpoint placement on the outlined frontal sinus with an overlay measurement aid (yellow lines); (**a**) line placed on the innermost sinus contour but not at the outermost intersection with the yellow ray, (**b**) line extended to the outermost intersection of the yellow ray and the blue sinus outline, following the method of Cox et al. [16].

**Figure 3:**
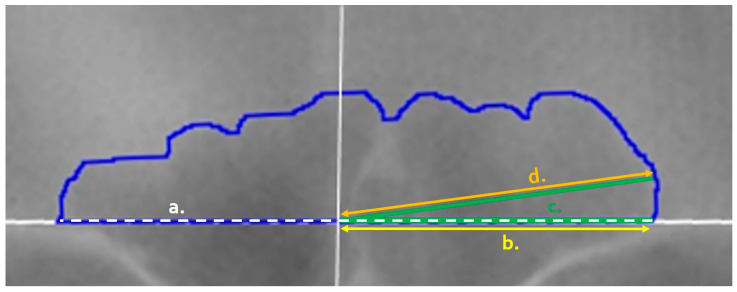
Variables of the frontal sinus as recorded in ImageJ: (**a**) Baseline Length (white dashed line) captures the sinus outline base; (**b**) Right Baseline Length (yellow, offset) captures the right-side of the image baseline from the origin (marked by a solid white vertical line); (**c**) the Angle Perimeter (green) is drawn from the right side of the image baseline to the origin (fixed arm, dashed) and to the inner contour of the blue sinus outline along the specified degree (movable arm, solid green); and (**d**) the Line Length (orange) is calculated via the subtraction of the right baseline length (yellow) from the angle perimeter length (green).

Table 1 outlines the items that were collected/placed on each outlined image regardless of the mode of collection. Following Cox et al., lines were placed at 3° intervals from the origin to the inner sinus contour in a counter-clockwise fashion from 3° to 177° (see Figure 2 and the details below). These measurements were used to calculate the following for the technical portion of this study (see the statistical analyses): the placement of the origin, the precision of each angle degree, and the precision of each line length. The placement of the origin was assessed as a midline ratio, calculated as the right baseline length (Figure 3, yellow line) divided by the total baseline length (Figure 3, white dashed line). The angle degree was directly recorded by ImageJ when measuring each placed angle perimeter (Figure 3, green line). However, when using the angle function, ImageJ records the combined lengths of both lines that compose the angle: i.e., the fixed arm (the horizontal baseline of the angle) plus the moveable arm (the line from the origin to the sinus outline). To calculate the length of the line (Figure 3, orange line) from the origin to the sinus outline (as needed in the TDM), the right baseline length was subtracted from the total perimeter of the angle. A final variable—the time required to conduct the TDM per image—was recorded using an external device. Not all images have the time recorded, and if there was a problem or other interruption during the technique, the paused or extended times were removed as inaccurate representations of the method.

#### 2.2.1. General, Freehand Mode Procedure

The exact steps for obtaining the variables utilized in the Total Difference Method varied depending on which data collection mode was used (e.g., Freehand vs. Overlay vs. Semi-Auto Modes). The procedure used in the Freehand Mode will be outlined first, as the remaining modes stem from it. For each radiograph, the following general technique was followed in ImageJ, adhering to the original method [16] description as closely as possible. Step-by-step instructions on how to conduct this procedure in ImageJ are provided in the TDM Manual, available for download on GitHub (https://github.com/jcampbelljess/FrontalSinus_TD_macros/blob/main/TDM_Manual.pdf (accessed on 29 June 2022)).

**Figure 4:**
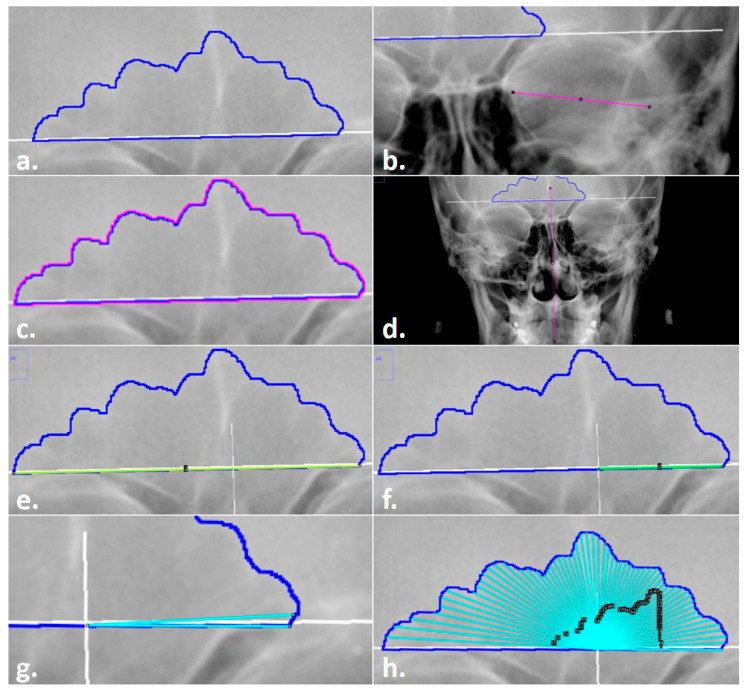
Steps in the Total Difference Method: (**a**) sinus outline on a radiograph; (**b**) measurement of orbital breadth for scaling; (**c**) highlighted sinus outline to be measured for the area and perimeter; (**d**) line drawn through midline landmarks to identify the origin of the sinus baseline; (**e**) line drawn for the baseline measurement; (**f**) line drawn from the origin for the right (side of the image) baseline measurement; (**g**) preliminary angle drawn at 3°; (**h**) completed angles with black ROI Manager numbered labels for editing, if required.

1. A radiograph .tiff file with an existing sinus outline was opened in ImageJ (Figure 4a).
2. Each radiograph was scaled to an orbital breadth at 39.49 mm using the *line tool* (Figure 4b). Specifically, the orbital breadth measurement was taken on the left orbital cavity from the dacryon to the ectoconchion. If the left orbital cavity was obscured, the measurement was taken on the right. The collection of orbital breadths differs from that of Cox et al. [16] but was necessary, as radiographic protocols can vary widely even within institutions, hindering accurate size measurements. As size is an important component of this specific technique, images were scaled using the orbital breadth (OBB) averaged from all the groups in Howell’s dataset OBB ($\bar{x}$ = 39.49 mm, s = 2.024) [32].
3. The previously traced sinus outline was highlighted using the *magic wand tool*, and the measurement of the area (inside of the outline) and the perimeter (outer shape) was collected to obtain an overall measurement of sinus size and to serve as a secondary check that the repeated measurements of the images were scaled (Figure 4c). This measurement was recorded in the Region of Interest (ROI) Manager available in ImageJ for measurement at the end of the technique. (The ROI Manager records all objects placed on an image and enables users to review or edit the placement at any time thereafter; see the supplementary materials for details on how to implement this feature.)
4. A point of origin was added at the midline of the skull using the *line tool* (Figure 4d). Following Cox et al. [16], this point was determined by drawing a vertical line through the skull using as many midline anatomical landmarks as possible (including nasion, anterior nasal spine, prosthion, etc.). The point of the midline that intersected the sinus baseline was determined as the origin, from which the right baseline and the 59-line lengths were collected (see steps 5 and 7).
5. The *line tool* was used to trace the total baseline and the right-side baseline of the inferior border of the sinus (Figure 4e,f). The baseline represents the entire baseline of the sinus, from the left to the right sides. Per the original method, the right baseline refers to the right side of the radiograph, not the cranium, and was measured from the origin to the right terminus of the baseline. The lengths for each were recorded in the ROI Manager.
6. The distances of 59 lines from the origin to the sinus outline at 3° intervals were collected using the *angle tool* (Figure 4g,h). First, the initial end of the angle was placed on the right terminus of the outline baseline; then, the created line was positioned on the origin to form the fulcrum; finally, the line was extended to the 3° intercept with the sinus outline. When drawing lines, three directives were followed per the original method. First, the separate lines were drawn to the internal border of the sinus outline. Second, the lines were collected in a counterclockwise fashion, starting on the right side of the image. Third, in the event a 3° ray crossed the outline more than once, the outermost intersection was used (see Figure 2). All 59 lines were recorded separately in the ROI Manager. At this point, if any lines needed to be adjusted by length or angle, they could be selected in the ROI Manager and the endpoints adjusted in the image.
7. Upon completion, all variables in the ROI Manager (i.e., sinus area, sinus perimeter, total baseline, right baseline, and the 59 lines/angles) were then selected, measured, and recorded for analysis in an excel file before clearing the workspace and opening the next image.

#### 2.2.2. Overlay Mode Procedure

The Overlay Mode procedure utilizes a measurement aid that can be downloaded from https://github.com/jcampbelljess/FrontalSinus_TD_macros (accessed on 29 June 2022). Additional details regarding the use of the overlay and step-by-step instructions are available in the TDM Manual file available on the GitHub repository for this method. This mode followed the general Freehand Mode procedures through Step 4, where the origin (or midline of the skull) was marked. The modified steps are below.

**Figure 5:**
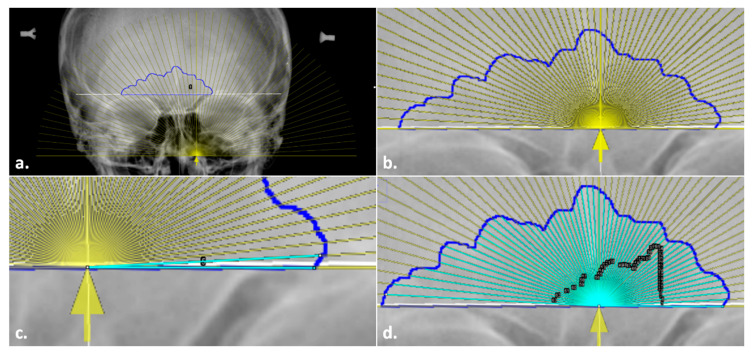
Placing the overlay in the modified steps in the Total Difference Method: (**a**) insertion of a semi-transparent overlay (yellow) onto the radiograph with the sinus outline and origin marked; (**b**) alignment of the overlay baseline and origin arrow (yellow) with the sinus outline (blue) and midline (underlying white solid line); (**c**) preliminary angle drawn at 3°; (**d**) completed angles with black ROI Manager numbered labels for editing, if required.

5. The *line tool* was used to trace the total baseline and the right-side baseline of the inferior border of the sinus, in the same manner as described in the general technique. However, in addition, the angle of the baseline was recorded from the command bar for the next steps.
6. In instances where the original radiograph was tilted, the radiographic image was rotated to align and level the supraorbital line to a 0° angle using the *Transform submenu, Rotate* command in ImageJ. The image could then be rotated manually or by entering the baseline angle recorded from Step 5 (above). This step was required for accuracy in the placement of the measurement aid and the positioning of the subsequently traced 3° lines.
7. A semi-transparent measurement aid was then overlaid onto the sinus outline. To do so, the overlay file (“Overlay.tif”; available on GitHub) was opened in a separate window and inserted onto the radiograph at 25% transparency using the *Overlay submenu*, *Add Image* command in ImageJ (Figure 5a). The overlay was then added to the ROI Manager to allow for repositioning and fine-tuned image rotation for alignment, as well as to enable the ability to toggle on/off. When placing the overlay, both the baselines and the marked origins of the outline and the measurement aid were aligned horizontally and vertically, respectively (see Figure 5b).
8. Once the measurement aid was placed, the *angle tool* was used to obtain the 59 lines from the origin to the sinus outline (see the general technique in Step 6). Unlike the Freehand Mode, the lines were placed by tracing the overlay as a guide instead of manually determining the 3° placement (Figure 5c,d). This process still followed the general rules outlined above (e.g., the lines went to the inside of the contour in a counter-clockwise fashion). Upon completion of the 59 lines, Step 7 in the general technique was followed as before to record all the variables in an excel file.

#### 2.2.3. Semi-Auto Mode Procedure

The Auto-Mode procedure includes a semi-automated macro that is managed through ImageJ. Note that it is not completely automated regarding the collection of the variables; the user is still required to visually identify landmarks for scaling, place the origin and overlay, and draw the baselines and the 59 lines to the appropriate sinus edges. However, the script walks the user through the steps listed above using pop-up prompts, including the added alignment of the overlay, before generating and saving the data, clearing the workspace, and opening the next image in the assigned folder. Aside from starting the macro and following the prompts, all of the steps are similar to the Overlay Mode procedures.

The overlay, zipped ROI starter file, and script written in FIJI using Javascript are available on the GitHub repository for this method. The script requires some modifications by the user prior to implementation, primarily by specifying the names of the folders where the radiographs are stored, what the images should be scaled by, and the location to save the .csv output that is generated at the end of every cycled image. Detailed instructions on how to use these files are provided in the TDM Manual, also available on GitHub.

### 2.3. Statistical Analyses

In addition to time, five variables were used to test the mean error of the technical method: midline placement, the bias and inaccuracy of the angle placement, and the bias and inaccuracy of the line lengths. The bias and inaccuracy (precision) from the angles and line lengths are the primary measures of mean error. Bias is the difference between the observed and expected values and assesses how far the estimated value is from the true value. Inaccuracy, better referred to as precision, is the absolute difference, which reflects the measure of the similarity of each estimate to another but not to the true value. When accessed together, bias and precision capture the accuracy of a method.

**Figure 6:**
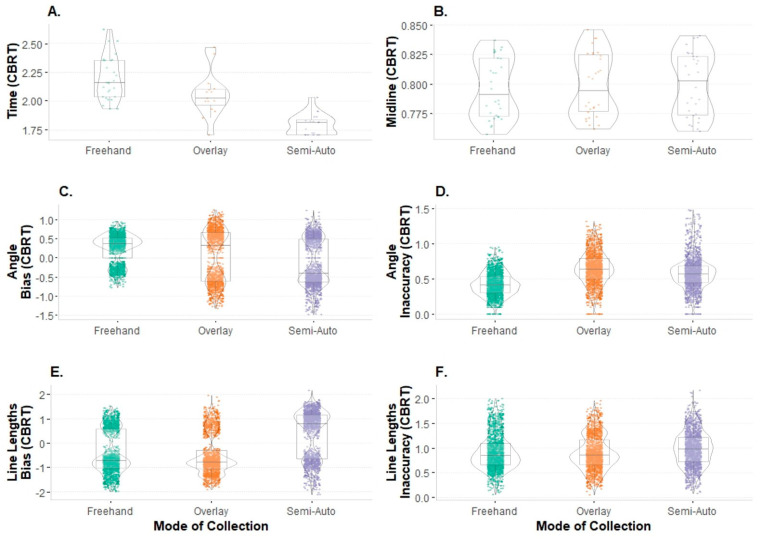
Violin plots of dataset by mode, transformed and representing distributions and underlying box-and-whisker plots visualizing summary statistics: (**A**) time; (**B**) midline ratio; (**C**) bias in the angle placement; (**D**) inaccuracy in the angle placement; (**E**) bias in the line lengths; (**F**) inaccuracy in the line lengths (CBRT = cube root.).

Exploratory data analyses were performed to assess the data for entry errors, normalcy, and the presence of outliers. Time and midline ratios were evaluated as single summary points per image and mode event (*n* = 90), and the bias and inaccuracy for the angle placement and line lengths were evaluated using all of the 59 lines per image and mode event (*n* = 5310 data points). Shapiro–Wilks tests showed that the variables of interest, when considered by mode, were non-normal and required transformation. Since two of the variables contained negative values (e.g., bias is the directional error), a cube-root transformation was applied. The transformed data were used for application in the repeated measures analyses of variances (RM ANOVAs) described below. Of the six variables of interest, all of them except for the midline ratio retained outliers. Extreme outliers (exceeding three times the interquartile range) that might greatly influence the results were identified and subsequently removed. This removed 1 of 5310 data points, resulting in a final sample of 5309 data points prior to subsetting for inter- and intraobserver error testing. The distributions of the complete transformed dataset with the outliers removed are visualized by mode with violin plots, with associated summary statistics for each mode (Figure 6).

The data were analyzed using RM ANOVA tests to accommodate repeat observations within the same dataset. A one-way RM ANOVA was used to evaluate the three modes on time efficiency. A two-way RM ANOVA was used to evaluate intra- and interobserver errors in the midline placement, errors in the angle placement, and deviations in the line lengths for the three modes by round of observation (intraobserver) or observer (interobserver). Intraobserver error was tested for one observer using all three modes, with data collection events spanning a minimum of two weeks apart. Interobserver error was tested using all first-round measurements in the three modes for two observers. When significant differences were identified in the RM ANOVA, post hoc analyses consisted of pairwise comparisons with a Holm family-wise error rate (FWER) correction. All analyses were completed in R [27].

## 3. Results

### 3.1. Intraobserver Error

There was no difference in any of the four measures of the mean error between the rounds of data collection when the modes were analyzed at the degree level. Two-way RM ANOVAs indicated no difference for the intraobserver placement of the midline or the inaccuracy of the angle placement across the three modes between rounds one and two (Table 2). Since the two-way model was overfitted for the midline, a one-way model was used; without taking the mode of collection into account, this model indicated no difference between the rounds of data collection (F_1,9_ = 0.289, *p* = 0.604). The mean placement on the origin midline point ratio ranged from 0.51 to 0.53 (± ~0.05) in all three modes and by observation round.

The bias (directional error) in the angle placement was different between rounds for all three modes (F_2,18_ = 4.036, *p* = 0.036). Pairwise tests showed that all three modes retained significance with a Holm FWER correction. The differences between rounds were approximately similar for both the overlay (*p.adj* < 0.0001) and the Semi-Auto Mode (*p.adj* < 0.0001), but the Freehand Mode had the smallest margin of error (*p.adj* < 0.001).

The RM ANOVA for the accuracy in the line lengths indicated a difference between the rounds for the three modes in terms of both bias (F_2,18_ = 11.330, *p* = 0.001) and inaccuracy (F_2,18_ = 5.135, *p* = 0.017), though not when the line lengths were segregated by degree. In pairwise testing, all three modes retained adjusted significance in terms of both bias and inaccuracy. The Freehand Mode had the lowest bias and inaccuracy overall, and the Semi-Auto Mode had the most drastic improvement from round one to round two.

### 3.2. Interobserver Error

The two-way RM ANOVA indicated that there were no differences between observers for the placement for the midline ratio, the bias in the angle placement, and the bias in the line lengths across the three modes (Table 3). However, a significant difference between observers within modes was found for inaccuracy in both the angle placement (F_2,18_ = 5.704, *p =* 0.012) and the line lengths (F_2,18_ = 8.069, *p =* 0.003). For inaccuracy in the angle placement, pairwise comparisons with a Holm correction identified that the difference between observers occurred in the Freehand Mode (t_1086_ = 15.744, *p <* 0.0001) and the Overlay Mode (t_1149_ = −6.792, *p <* 0.0001). Pairwise comparisons identified differences in all three modes for inaccuracy in the line lengths, which retained significance after the Holm correction was applied (Freehand Mode, Overlay Mode, *p <* 0.0001; Semi-Auto Mode, *p <* 0.01).

Since a difference in the line lengths could directly impact the accuracy rates of using this method for quantifying an antemortem-postmortem match, and the error terms were approximated, the transformed line lengths were evaluated to clarify the differences between the observers in each mode. The two-way RM ANOVA was not significant (F_2,18_ = 0.788, *p* = 0.47), though a pairwise post hoc test was still performed to inspect whether the results for the line length inaccuracy were tangible in the individual line lengths. Only the Freehand Mode retained significance with the Holm correction (t_1178_ = 2.561, *p <* 0.05), and there was no difference in the line lengths in the Overlay Mode or the Semi-Auto Mode between the observers.

### 3.3. Time

The RM ANOVA revealed that time was significantly different between the modes of collection (F_2,18_ = 52.71, *p <* 0.0001) for all available observations. Pairwise comparisons revealed that the Semi-Auto Mode ($\bar{x}$ = 5.88 min, s = 0.91) was more quickly performed than both the Freehand Mode (t_41.1_ = 9.862, *p.adj* < 0.0001) and the Overlay Mode (t_18.6_ = 4.795, *p.adj* < 0.001). There was not a significant difference between the times for the Freehand Mode ($\bar{x}$ = 10.90 min, s = 2.86) and the Overlay Mode ($\bar{x}$ = 8.91 min, s = 2.61).

Since each event was independent and time was not recorded for all observations, resulting in an unequal sample size for each mode, time was analyzed using all available observation points. When the time was segregated by observer, pairwise comparisons noted a difference in only the Freehand Mode (t_16.9_ = −5.11, *p.adj* < 0.001). This was also true when the data were segregated by the round of collection (Freehand Mode (t_23.7_ = 4.03, *p.adj* < 0.01). The overall time improvement in the Freehand Mode was 1.36 min from the first round of data collection to the second.

## 4. Discussion

The goal of the current study was to conduct an initial validation of the Total Difference Method, a technique developed by Cox et al. [16], using a small sample (*n* = 10 individuals). In addition to testing the original TDM (referred to as the Freehand Mode in this study), the current study tested two modifications with the inclusion of a measurement aid (Overlay Mode) and a semi-automated macro (Semi-Auto Mode). These modifications were implemented to increase efficiency without impeding repeatability. Overall, the current study indicated high intra- and interobserver repeatability using the TDM, regardless of mode. Specifically, the placement of the midline (to obtain the origin) and inaccuracy in the angle placement and the line lengths are repeatable, and the method can be replicated reliably regardless of the data-collection mode.

Importantly, the current study also found that the addition of the measurement aids in the Overlay Mode and Semi-Auto Mode did not hinder the repeatability of the TDM. The placement of the measurement aid in both the Overlay Mode and Semi-Auto Mode was consistent and repeatable within and between observers. The use of the overlay increased repeatability in some instances. When comparing the modes between rounds, the greatest improvements were noted with the use of the measurement aid in either the Overlay Mode or the Semi-Auto Mode, suggesting that applying this method without the use of an overlay introduces error that could otherwise be controlled with a measurement aid. This conclusion was supported by the bias and inaccuracy in the line lengths, which were identified as significant after the Holm correction was applied.

Of additional interest is that the use of the measurement aid (in both the Semi-Auto Mode and Overlay Mode) increased efficiency in the TDM. Specifically, this study indicates that the use of the semi-automated macro (Semi-Auto Mode) significantly decreased the amount of time needed to collect data per individual compared to the Overlay Mode and Freehand Mode. Further, due to the numerous steps in this technique and the learning curve for ImageJ, it was expected that time would improve with familiarity. This expectation was realized in the Freehand Mode and the Overlay Mode. There was a significant difference in time between the observers as well; this is likely attributable to the first observer’s training and familiarity with the specific method conducted during the development phase of this research.

The amount of time needed to implement a method in forensic identifications or related basic science research is an important consideration. Indeed, the complexity of a method—including the time it takes to conduct it—is an important practical consideration that has been discussed in the forensic literature, e.g., [33,34,35,36,37]. While time is often discussed, it is not always directly collected and tested across methods [36]. Here, we encourage future tests of validation, particularly of newly devised or modified methods, to collect this informative variable. As mentioned in the current study, the use of the semi-automated macro significantly decreased the amount of time needed to perform the TDM on each individual. However, the time needed to learn and become comfortable with the macro may in itself be daunting to individuals not familiar with ImageJ or macros in general—especially if it is only used sparingly for an individual identification with a single antemortem and postmortem image. With this in mind, the use of the macro would be most beneficial to researchers intending to apply the TDM to larger (*n* > 10), more diverse datasets (*n* > 10) for future validation studies. When conducted, we encourage future researchers to share these data—either as published data, as done here, or through archives such as the RADid Resource developed by Christensen and Hatch [38]. As the current study showed acceptable levels of interobserver error, these data can be combined to serve as databases for studies on sinus variation and population frequencies and/or as a comparative database for actual identification procedures, benefitting forensic practitioners who may use this method. Indeed, the call for comparative databases has been a common notion in the forensic identification literature, particularly for the frontal sinus [17,38]. As such, research investigating and validating the success rates of using this method for personal identification is currently ongoing on larger, more diverse samples, with the implementation of the measurement aid validated in the current study. Such studies would also allow for better discussion and interpretation regarding identification error rates and the comparison of potential benefits versus limitations when utilizing the TDM for personal identification.

A final benefit of the current study is the application of the TDM, regardless of mode, in a freely-available resource: ImageJ. Step-by-step methods for each of the three modes are provided in the TDM Manual available on the GitHub repository. We hope that these directions allow for more transparency and repeatability in collecting the data for this method, as well as reach more investigators/researchers who may not have access to the licensed software, such as Adobe Photoshop^®^ CS2 [31], utilized in the original method.

## 5. Conclusions

The current study indicates that the Total Difference Method for quantifying the frontal sinus shape is both reliable and repeatable between and within observers. The efficiency and repeatability are further increased with the use of the measurement aid, which was developed for and implemented in this study using freely accessible resources (https://github.com/jcampbelljess/FrontalSinus_TD_macros (accessed on 29 June 2022)). However, there is a learning curve for practitioners unfamiliar with ImageJ and/or editing Javascript. Practice with both of these, as well as applying the steps of this method, will further reduce error and allow for additional testing of the TDM on much larger samples. Such research would be important not only to continue validation testing of this identification method but also to increase comparative databases when using this method in forensic practice.

## Figures and Tables

**Table 1:** Measurements recorded during the data collection.

Measurement	Definition	Purpose
**Supraorbital Margin Line**	A line drawn between the most superior edge of the left and right orbital margins.	Demarcates the inferior border of the frontal sinus.
**Left Orbital Breadth**	As defined by Howells, 1973, taken from the ectoconchion to the dacryon on the anatomical left orbital cavity.	Without a scale included in the radiograph, this scales all the images in the sample to a standardized size.
**Sinus Area/Perimeter**	The area perimeter of the sinus, as traced by the ImageJ magic wand.	Backup check for the scaling parameter in the event the image has to be re-analyzed or there is a question of whether a scale was applied.
**Baseline Length**	A line tracing the inferior border (on the supraorbital line) of the frontal sinus.	(A) Backup check for the scaling parameter in the event the image has to be re-analyzed. (B) Used for the midline ratio.
**Midline/Origin**	The intersection of the inferior border of the sinus (on the supraorbital line) and a vertical line drawn through visible facial midline points.	The fulcrum for all 59 angles.
**Right Baseline Length**	A line from the origin to the end of the sinus baseline, drawn on the right side of the image (right terminus), following Cox et al. [16].	(A) Backup check for the origin placement parameter in the event the image has to be re-analyzed. (B) Length of the fixed arm of the subsequently drawn angles. (C) Used for the midline ratio.
**Angle**	A three-point line from the right terminus, to the origin, to the inner sinus contour. Repeated 59 times at every 3° around the sinus.	Measures the distance (line length) from the origin to the inner sinus contour (movable arm) after the right baseline length is subtracted (fixed arm).
**Time**	Recorded in minutes and seconds.	Evaluates the efficiency.

**Table 2:** Intraobserver error summary statistics and the results of two-way (round*mode) repeated measures analysis of variance (RM ANOVA) across the three modes using transformed data.

Variables		Freehand			Overlay			Semi-Auto			RM ANOVA	
		*n*	*mean*	*sd*	*n*	*mean*	*sd*	*n*	*mean*	*sd*	*F*	*p*
**Midline Ratio**		20	0.51	0.05	20	0.51	0.05	20	0.52	0.05	0.289 *	0.604
*Observation rounds*	*1*	10	0.51	0.05	10	0.51	0.05	10	0.52	0.05		
	*2*	10	0.51	0.04	10	0.52	0.05	10	0.53	0.05		
**Bias** °**angle placement**		20	0.14	0.15	20	0.00	0.24	20	−0.11	0.31	4.036	**0.036**
*Observation rounds*	*1*	10	0.10	0.03	10	0.09	0.22	10	0.00	0.20		
	*2*	10	0.19	0.20	10	−0.09	0.24	10	−0.22	0.37		
**Inaccuracy** °**angle placement**		20	0.17	0.15	20	0.31	0.15	20	0.28	0.26	0.044	0.957
*Observation rounds*	*1*	10	0.13	0.04	10	0.31	0.15	10	0.26	0.14		
	*2*	10	0.21	0.20	10	0.32	0.16	10	0.31	0.34		
**Bias** **line length**, **mm**		20	−0.13	0.44	20	−0.39	0.71	20	0.45	1.20	11.330	**0.001**
*Observation rounds*	*1*	10	−0.28	0.35	10	−0.75	0.79	10	1.25	1.06		
	*2*	10	0.02	0.48	10	−0.04	0.39	10	−0.36	0.69		
**Inaccuracy** **line length**, **mm**		20	0.57	0.26	20	0.64	0.56	20	1.00	0.81	5.135	**0.017**
*Observation rounds*	*1*	10	0.50	0.25	10	0.83	0.74	10	1.39	0.87		
	*2*	10	0.63	0.26	10	0.45	0.18	10	0.61	0.52		

Note: this table is presented using the transformed data. * indicates one-way RM ANOVA results; bold *p*-values indicate ≤ 0.05.

**Table 3:** Interobserver error summary statistics and the results of two-way (round*mode) repeated measures analysis of variance (RM ANOVA) across the three modes using transformed data.

Variables		Freehand			Overlay			Semi-Auto			RM ANOVA	
		*n*	*mean*	*sd*	*n*	*mean*	*sd*	*n*	*mean*	*sd*	*F*	*p*
**Midline Ratio**		20	0.51	0.05	20	0.51	0.05	20	0.51	0.05	2.763	0.090
*Observer*	*1*	10	0.51	0.05	10	0.51	0.05	10	0.52	0.05		
	*2*	10	0.50	0.05	10	0.51	0.05	10	0.50	0.05		
**Bias** °**angle placement**		20	0.06	0.04	20	0.07	0.37	20	−0.07	0.29	0.202	0.819
*Observer*	*1*	10	0.10	0.03	10	0.09	0.22	10	0.00	0.20		
	*2*	10	0.03	0.02	10	0.06	0.49	10	−0.14	0.35		
**Inaccuracy** °**angle placement**		20	0.09	0.05	20	0.39	0.22	20	0.29	0.21	5.704	**0.012**
*Observer*	*1*	10	0.13	0.04	10	0.31	0.15	10	0.26	0.14		
	*2*	10	0.05	0.03	10	0.48	0.26	10	0.32	0.27		
**Bias** **line length**, **mm**		20	−1.07	1.47	20	−1.08	1.23	20	0.96	1.56	0.844	0.446
*Observer*	*1*	10	−0.28	0.35	10	−0.75	0.79	10	1.25	1.06		
	*2*	10	−1.87	1.75	10	−1.40	1.53	10	0.66	1.96		
**Inaccuracy** **line length**, **mm**		20	1.34	1.31	20	1.39	0.98	20	1.56	1.02	8.069	**0.003**
*Observer*	*1*	10	0.50	0.25	10	0.83	0.74	10	1.39	0.87		
	*2*	10	2.18	1.42	10	1.96	0.88	10	1.74	1.16		

Note: this table is presented using transformed data. Bold *p*-values indicate ≤0.05.

## Data Availability

The radiographs are publicly available online through the AAOF. Other data are available on request from the authors and/or on GitHub at https://github.com/jcampbelljess/FrontalSinus_TD_macros (accessed on 29 June 2022).

## Supplementary Materials

All the materials, code, and data used in this publication are freely available for download on GitHub at https://github.com/jcampbelljess/FrontalSinus_TD_macros (accessed on 26 June 2022).
